# Cortical activity during sensorial tactile stimulation in healthy adults through Vojta therapy. A randomized pilot controlled trial

**DOI:** 10.1186/s12984-021-00824-4

**Published:** 2021-01-21

**Authors:** Ismael Sanz-Esteban, Roberto Cano-de-la-Cuerda, Ana San-Martín-Gómez, Carmen Jiménez-Antona, Esther Monge-Pereira, Cecilia Estrada-Barranco, José Ignacio Serrano

**Affiliations:** 1 Department of Physiotherapy. Physical Therapy and Health Research Group, Faculty of Sport Sciences, Universidad Europea de Madrid, Madrid, Spain; 2 Department of Physical Therapy, Occupational Therapy, Physical Medicine and Rehabilitation, Faculty of Health Sciences, Rey Juan Carlos University, Avenida de Atenas s/n, 28922 Alcorcón, Madrid Spain; 3 Neural and Cognitive Engineering Group (gNeC), Automation and Robotics Center, Spanish National Research Council (CSIC-UPM), Madrid, Spain

**Keywords:** Brain activity, Cortical activation, Electroencephalography, Reflex locomotion, Tactile stimulation, Vojta therapy

## Abstract

**Background:**

Brain’s is stimulated by Vojta Therapy through selected body areas activating stored innate motor programs which are exported as coordinate movement and muscle contractions to trunk and limbs. The aim of this pilot study is to know the responses at cortical level to a specific tactile input, assessed by electroencephalography (EEG), compared to a sham stimulation, in healthy subjects.

**Methods:**

A randomized-controlled trial was conducted. Participants were randomly distributed into two groups: a non-specific tactile input-group (non-STI-group) (n = 20) and a Vojta specific tactile input-group (V-STI-group) (n = 20). The non-STI-group was stimulated in a non specific area (quadriceps distal area) and V-STI-group was stimulated in a specific area (intercostal space, at the mammillary line between the 7th and 8th ribs) according to the Vojta therapy. Recording was performed with EEG for 10 min considering a first minute of rest, 8 min during the stimulus and 1 min after the stimulus. EEG activity was recorded from 32 positions with active Ag/AgCl scalp electrodes following the 10–20 system. The continuous EEG signal was split into consecutive segments of one minute.

**Results:**

The V-STI-group showed statistically significant differences in the theta, low alpha and high alpha bands, bilaterally in the supplementary motor (SMA) and premotor (PMA) areas (BA6 and BA8), superior parietal cortex (BA5, BA7) and the posterior cingulate cortex (BA23, BA31). For the V-STI-group, all frequency bands presented an initial bilateral activation of the superior and medial SMA (BA6) during the first minute. This activation was maintained until the fourth minute. During the fourth minute, the activation decreased in the three frequency bands. From the fifth minute, the activation in the superior and medial SMA rose again in the three frequency bands

**Conclusions:**

Our findings highlight that the specific stimulation area at intercostal space, on the mammillary line between 7 and 8th ribs according to Vojta therapy differentially increased bilateral activation in SMA (BA6) and Pre-SMA (BA8), BA5, BA7, BA23 and BA31 in the theta, low and high alpha bands in healthy subjects. These results could indicate the activation of innate locomotor circuits during stimulation of the pectoral area according to the Vojta therapy.

*Trial registration* Retrospectively registered. This randomized controlled trial has been registered at ClinicalTrials.gov Identifier: NCT04317950 (March 23, 2020).

## Introduction

Reflex Locomotion or Vojta Therapy was first used in 1959 for the rehabilitation of children with motor alterations and infants with a risk of cerebral palsy. Years later it was successfully applied to adults with neurological and motor alteration problems [1]. Two different locomotion complexes were described by Dr. Vojta, reflex creeping and reflex rolling, which are triggered through an adequate positioning and stimulation of the appropriate trigger zones [2]. A global reflex answer due this activation is evoked, containing innate motor programs related to locomotion patterns in human ontogenesis (rolling, crawling, walking). Brain is stimulated by Vojta Therapy activating stored innate motor programs which are exported as coordinate movement to trunk and limbs. With the so-called “Reflex locomotion”, Dr. Vojta developed a method which enable the access to those innate motor programs even when a damage is at the motor system or central nervous system [3].

Conventional rehabilitation techniques have been considered as bottom-up approaches as they act on the distal physical level (bottom) aiming at influencing the neural system (top) [4]. Under this paradigm, Vojta therapy proposes tactile stimulation in selected body areas, such as the chest, iliac spine, calcaneus, o humeral epicondyle, to activate innate motor programs in humans [5]. During this stimulation, contractions and movements patterns may be observed which may achieved motor behavior and postural control changes [6–9]. The application of these stimuli has been shown to generate neuromodulatory activity in subcortical structures such as putamen, brainstem, cerebellum or reticular formation [10, 11].

The Vojta method has been mainly developed to treat patients with brain damage following pattern generator theories for postural and gait control on the assumption that brain damage somehow inhibits without disrupting the stored movement patterns [4]. This bottom-up approach is assumed to be able to rehabilitate patients due to the mechanisms of neural plasticity. However, how these mechanisms are established is still unknown, despite existing accurate descriptions of the movement patterns induced by Vojta therapy. In our knowledge only three previous researches, one conducted by our research group [10] and another by Hok et al. [11, 12], address and deepen on the knowledge of these movement patterns using imaging techniques (functional Magnetic Resonance Imaging, fMRI). These studies relate tactile stimuli application with cortical and subcortical areas activation. Consequently, it is essential to investigate which cortical areas are activated during tactile sensory stimulation, which may provoke innate and automatic movement patterns to start up to establish therapy’s neurophysiological basis, firstly in healthy population.

Since the majority of rehabilitative methodologies nowadays applied are bottom-up, as Vojta therapy, they act on the physical level and expect for changes at the central neural system level, it is reasonable to have a better insight about the mechanisms that justify these type of approaches and to know the activation of brain areas related with different body stimuli [13]. In this context, studies are prompted to develop an evidenced-based model to understand Vojta therapy through specific proprioceptive and tactile stimulus in pectoral zone and their brain activation areas [14].

Among brain activity assessment approaches, the electroencephalographic (EEG) study of the behaviors of cortical oscillations related to movement provide an integral view of the mechanisms of brain organization, since movements generate electroencephalographic activity variations over cortical areas [15]. EEG signals are relevant, given their highly accurate temporal resolution and their suitability in clinical environments. EEG-based technologies allow real-time characterization of motor-related cortical activities to obtain predictive information regarding intended movement actions. Such information has proven to be valuable in providing motor function feedback at specific instant [16, 17]. With respect to Vojta therapy, EEG also allows studies of much longer stimulation periods than fMRI due to two reasons; first, it is much less sensitive to movement artifacts when the stimulation triggers them; second, the time of the therapist applying the stimulation in the fMRI room is very limited, but unlimited with EEG. In addition, the source power estimation by inverse-problem resolution techniques from the EEG signal provide more spatial resolution, concretely at a voxel level in the cortex, than conventional electrode-based EEG analysis, allowing a more accurate localization of activity differences.

Therefore, the aim of this pilot study is to know the responses at cortical level to a specific tactile input, assessed by EEG source analysis, compared to a sham stimulation, in healthy subjects. To our knowledge, this is the first study that has used EEG to assess brain activity during to tactile stimuli following Vojta´s approach on healthy subjects.

## Materials and methods

### Design

A randomized-controlled trial was conducted. Participants were randomly distributed into two groups using the EPIDAT 3.1 sofware: a non-specific tactile input-group (non-STI-group) (n = 20) and a Vojta specific tactile input-group (V-STI-group) (n = 20). None of the participants previously knew the groups or the area of the stimulus where it was going to be applied (participants blinded). The physiotherapist was the only one who knew the place of stimulation for the subjects. All assessments were recorded with an assessor-blinded.

Approval was obtained from the Ethics Committee of Rey Juan Carlos University, conforming to the Helsinki Declaration. This trial was retrospectively registered in ClinicalTrials with the register number NCT04317950 (March 23, 2020).

All participants received a document informing them of the study aims and signed an informed consent. The directives of the CONsolidated Standards of Reporting Trials (CONSORT) declaration [18] for non pharmacological RTCs were followed.

### Participants

Healthy subjects between 18 to 50 years old were recruited from Polibea Foundation at Tres Cantos (Madrid, Spain) where the study was to be developed. The study was designed with healthy patients in order to establish the neurophysiological bases of Vojta Therapy. Participant’s recruitment was made by e-mail, telephone calls and through an informative meeting. Once consent was signed by participants, a designated day and time to proceed with the study was established.

In total, 40 participants were initially recruited to take part in this study. Each participant received the corresponding intervention in a single 10-min session.

The inclusion criteria were healthy subjects without previous neurologic disease or any other pathology which may interfere in the intervention [19], between 18 to 50 years old [20, 21], non-alcoholic or drugs addition at the intervention moment [22, 23], be unaware of the foundations of Vojta therapy or its response of the stimuli after apply the therapy, non-pharmacological treatment which may affect nervous system functioning and may interfere in the intervention’s results.

The exclusion criteria were subjects who not fit the inclusion criteria, presence of any musculoskeletal alteration in the last 6 months [24], presence of any sensorial alteration [25, 26], presence of neurological disease or condition which may interfere at the intervention as pain, radiculopathy [24], presence of inflammatory illness or fever and pregnancy.

### Procedure

All participants were comfortably laid down on a stretcher on their back with eyes open, wearing a EEG cap during the intervention. They were asked to remain relaxed and still during the whole process. After a first minute of resting, V-STI-group received a continuous reflex locomotion stimulus during the next 8 min. On the contrary, non-STI-group received a continuous sham stimulus during the next 8 min. Both groups began and ended the intervention with an initial and final resting minute with no stimulus. Therefore, the interventions lasted 10 min during which the EEG signal was continuously recorded. Both sham and reflex locomotion stimuli were applied by a physiotherapist expert on Vojta therapy.

### Reflex locomotion and sham stimulation

The main difference between both stimuli (V-STI-group and non-STI-group) was the skin place of stimulation. V-STI-group was stimulated in a specific area (intercostal space, at the mammillary line between the 7th and 8th ribs) according to the Vojta therapy [27, 28] while the non-STI-group was stimulated in a non specific area (in the quadriceps distal area, 8 cm cranial from the superior angle of the patellar bone). This sham area was selected because it has no relation to any other known point within Vojta therapy or any other neurorehabilitation therapy [3].

All participants were placed in supine decubitus with a relaxed anatomical position. The head was rotated 30° at the same side of stimulation. Nevertheless, all of them were stimulated by an ipsilateral input. On the one hand, the V-STI-group was stimulated over the skin on the intercostal space, at the mammillary line between the 7th and 8th ribs. This stimulus was applied by the right thumb of the physiotherapist. A slight pressure with dorsal, cranial, and medial directional stimuli, toward the contralateral shoulder, during 8 min, according to Vojta theory was applied [27, 28]. On the other hand, the non-STI-group was stimulated in the non-specific area described above. Furthermore, the same direction and duration were performed in both groups on the right side.

### EEG acquisition and processing

An actiCHamp amplifier (Brain Vision LLC, NC, USA) was used to amplify and digitize the EEG data at a sampling frequency of 512 Hz. The EEG data were stored in a PC running Windows 7 (Microsoft Corporation, Washington, USA). EEG activity was recorded from 32 positions with active Ag/AgCl scalp electrodes (actiCAP electrodes, Brain Vision LLC, NC, USA) following the 10–20 system: F5, F3, F1, Fz, F2, F4, F6, FC5, FC3, FC1, FCz, FC2, FC4, FC6, C5, C3, C1, Cz, C2, C4, C6, CP5, CP3, CP1, CPz, CP2, CP4, CP6, P3, P1, Pz, P2. Ground and reference electrodes on were placed on Fz and on FCz, respectively.

EEG signal processing procedure was carried out using MATLAB functions (The Math- works Inc., Natick MA, USA), concretely the EEGLab toolbox [29]. The continuous EEG signal for each channel was artefact-corrected by the Artifact Subspace Reconstruction (ASR) algorithm [30], disabling all parameters except the high-pass filttran band width (0.25–0.75) and the burst repairing (kurtosis > 5). The signal was then band-pass filtered between 3 and 31 Hz with a Finite Impulse Response (FIR) filter (order 846). After that, channels beyond 5 standard deviations of the average channel kurtosis were automatically rejected and spherically interpolated. Next, Independent Component Analysis (ICA) was performed and artifact-related components according to the Multiple Artifact Rejection Algorithm (MARA) [31] (probability > 0.9), including eye blinks, were automatically removed.

For the intervention effect analysis, the continuous EEG signal was split into the first one-minute long segment (resting), the next 8-min long segment (stimulation), and last one-minute long segment (resting). The sLORETA algorithm [32] for source reconstruction was applied to each of these three segments. For the temporal evolution analysis, the continuous EEG signal was split into consecutive segments of 1 min. This one-minute duration was selected to get a relatively stable source power estimation based on duration of the triggered motor behaviors observed according to experienced therapists who participated in this study. The sLORETA algorithm was then applied to each one-minute segment of the processed signal, and the difference in source power of each one-minute segment with respect to the first minute segment was calculated. The sLORETA algorithm provided the source power for each of the 6239 voxels in which the algorithm divides the cortex, for six frequency bands: theta (4–7 Hz), low alpha (7–10 Hz), high alpha (10–13 Hz), low beta (13–18 Hz), mid beta (18–25 Hz) and high beta (25–30 Hz). The source power of each voxel was standardized by the average source power in each participant.

### Statistical analysis

For each group in each voxel and each frequency band, the significance of the differences in source power between the first resting minute and, the next overall 8-min period of stimulation and the last minute of resting were tested by statistical nonparametric mapping (SnPM) (33), using estimated t-test statistics assuming equal variances with 5000 permutations. This analysis was performed using the LORETA KEY software (KEY Institute for Brain-Mind Research, Zurich, Switzerland). Statistically significant difference was considered at a p-value < 0.05.

## Results

### Sociodemographic data

**Fig. 1:**
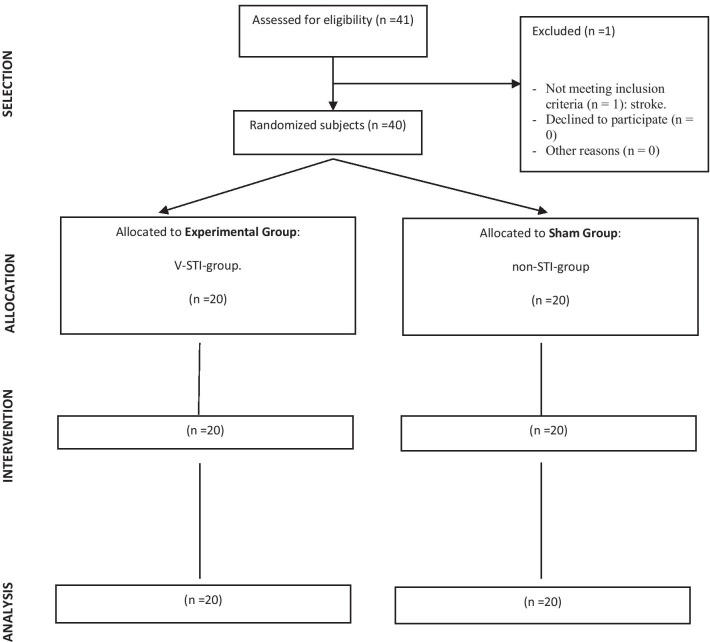
CONSORT flow diagram

The sample consisted of a total of 40 patients, 16 male and 24 female, of the 41 selected at the study onset (Fig. 1). One subject was excluded due to not meeting the inclusion criteria (stroke). The mean age of the whole sample was 30.3 ± 7.3. The mean age for the non-STI-group was 30.5 ± 5.67. The mean age for the non-STI-group V-STI-group was 30.1 ± 8.67. Sample features are summarized in Table 1.

**Table 1:** Sample features

Groups (n)	Age (years) Mean (± Standard deviation)	Male	Female
All sample	30.3 ± 7,3	16 40%	24 60%
Non-STI-group (20)	30.5 ± 5,67	9 45%	11 55%
V-STI-group (20)	30.1 ± 8,67	7 35%	13 65%

### Intervention effects on cortical activity

**Fig. 2:**
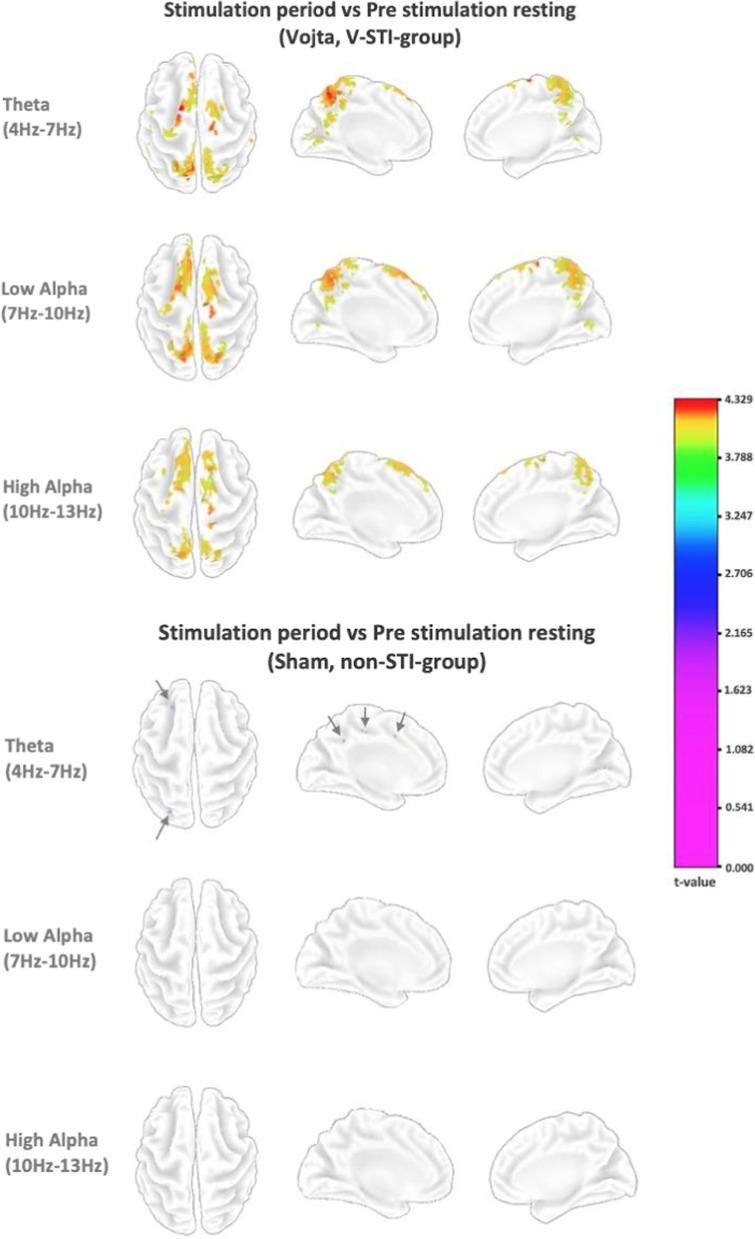
Colored t-values of voxels at different frequency bands that presented statistically significant differences between the first resting minute and the 8-min stimulation period for the two groups of study (top). The arrows on the top right point to the only colored parts in the sham stimulation group

**Fig. 3:**
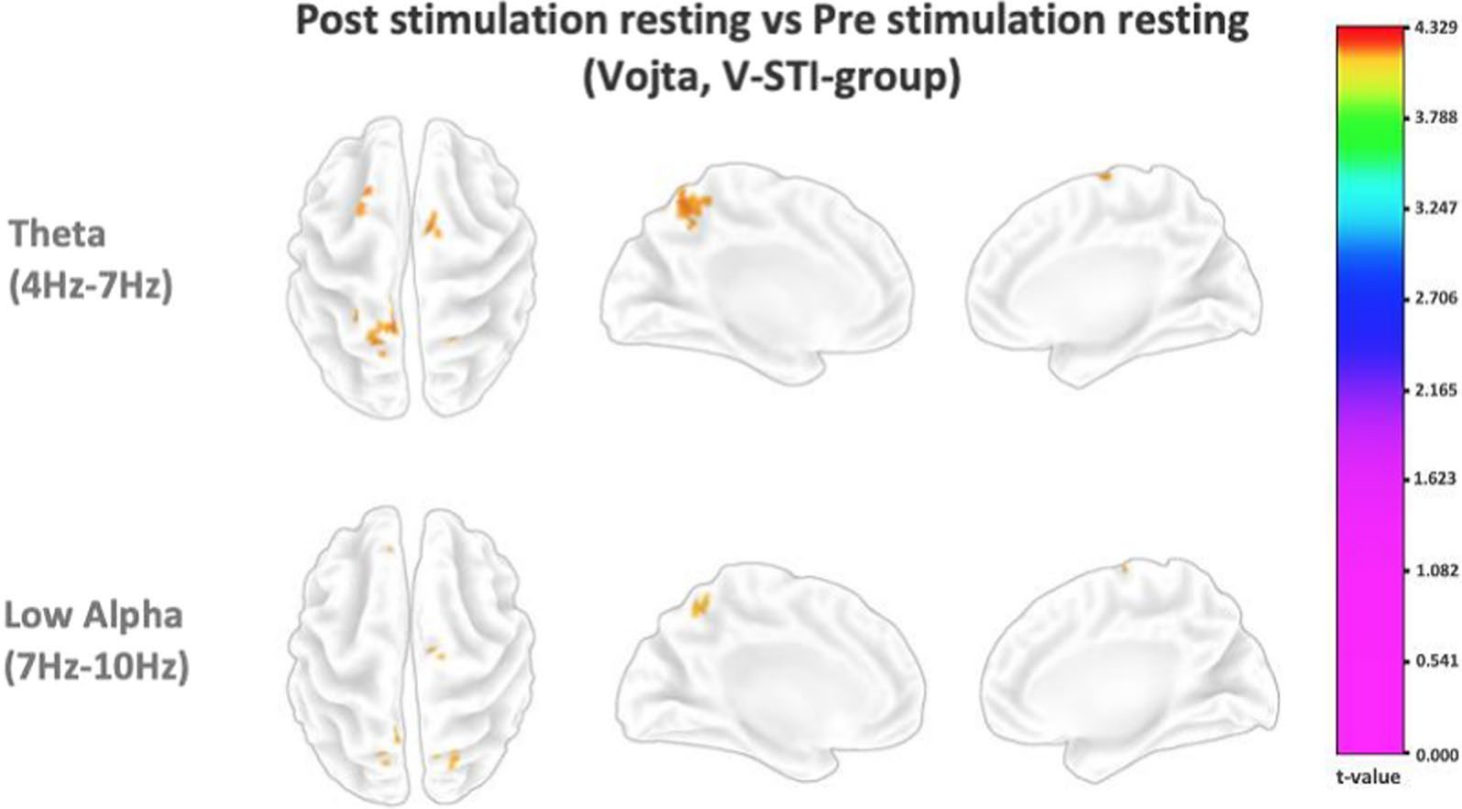
Colored t-values of voxels at different frequency bands that presented statistically significant differences between the first resting minute and last resting minute for the V-STI-group

Figure 2 shows the colored t-value only for the voxels that presented statistically significant differences between the first resting minute and the whole stimulation period (next 8 min) for both groups, and Fig. 3 between the first resting minute and last resting minute in the V-STI-group, at different frequency bands.

In the non-STI-group, the only statistically significant differences were found in the theta band, in very localized tiny areas of the left hemisphere in frontal, occipital and cingulate lobes (pointed by the arrows in Fig. 2). On the contrary, the V-STI-group showed statistically significant differences in the theta, low alpha and high alpha bands, bilaterally in the supplementary motor (SMA) and premotor (PMA) areas (Brodmann areas BA6 and BA8), superior parietal cortex (BA5, BA7) and the posterior cingulate cortex (BA23, BA31). In this group, some of the mentioned differences in BA6, BA7, and BA8 kept during the post intervention resting minute, specially in the theta band, although less pronounced and more narrowly located (Fig. 3). The non-STI-group did not present any statistical difference between the first and last resting minutes.

### Cortical activity evolution along the reflex locomotion stimulation period

**Fig. 4:**
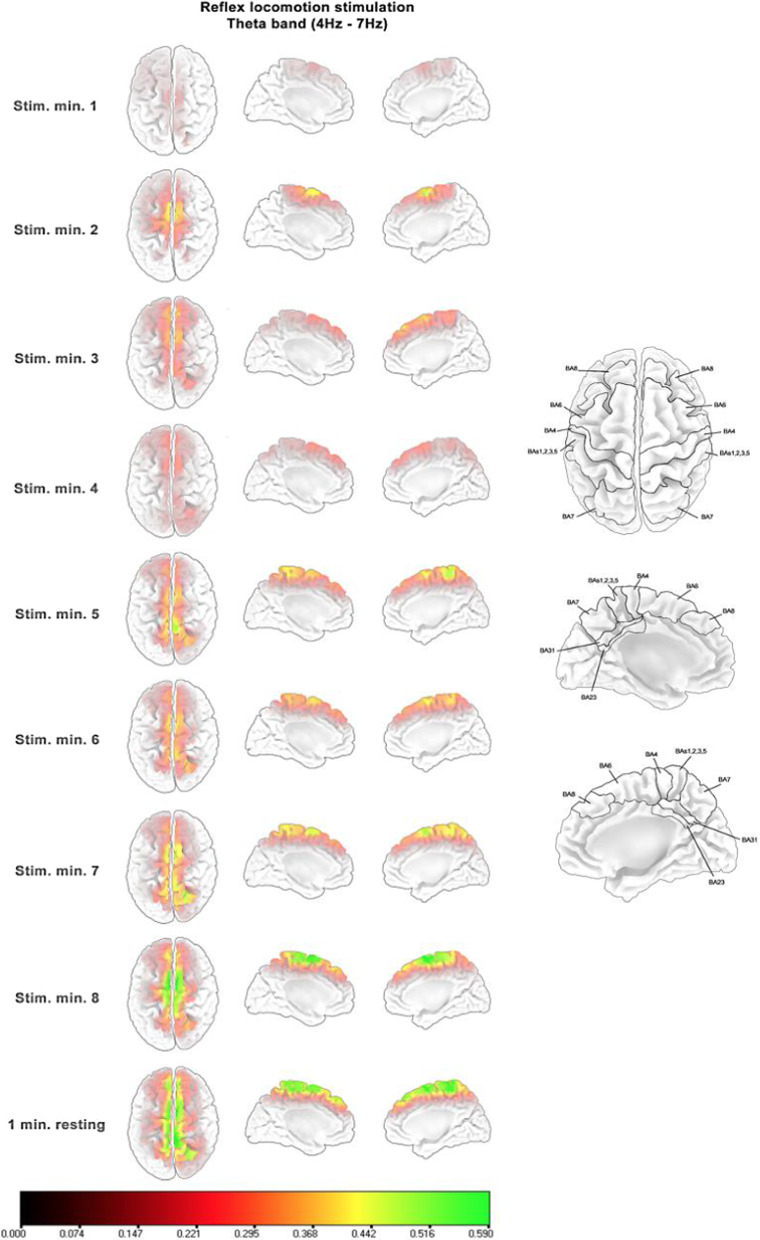
Standardized source power differences with respect to the first resting minute in consecutive minutes of reflex locomotion stimulation at theta band (4–7 Hz) for the V-STI-group. Implicated Brodmann areas are sketched over the cortex template on the right, according to the LORETA-KEY software

**Fig. 5:**
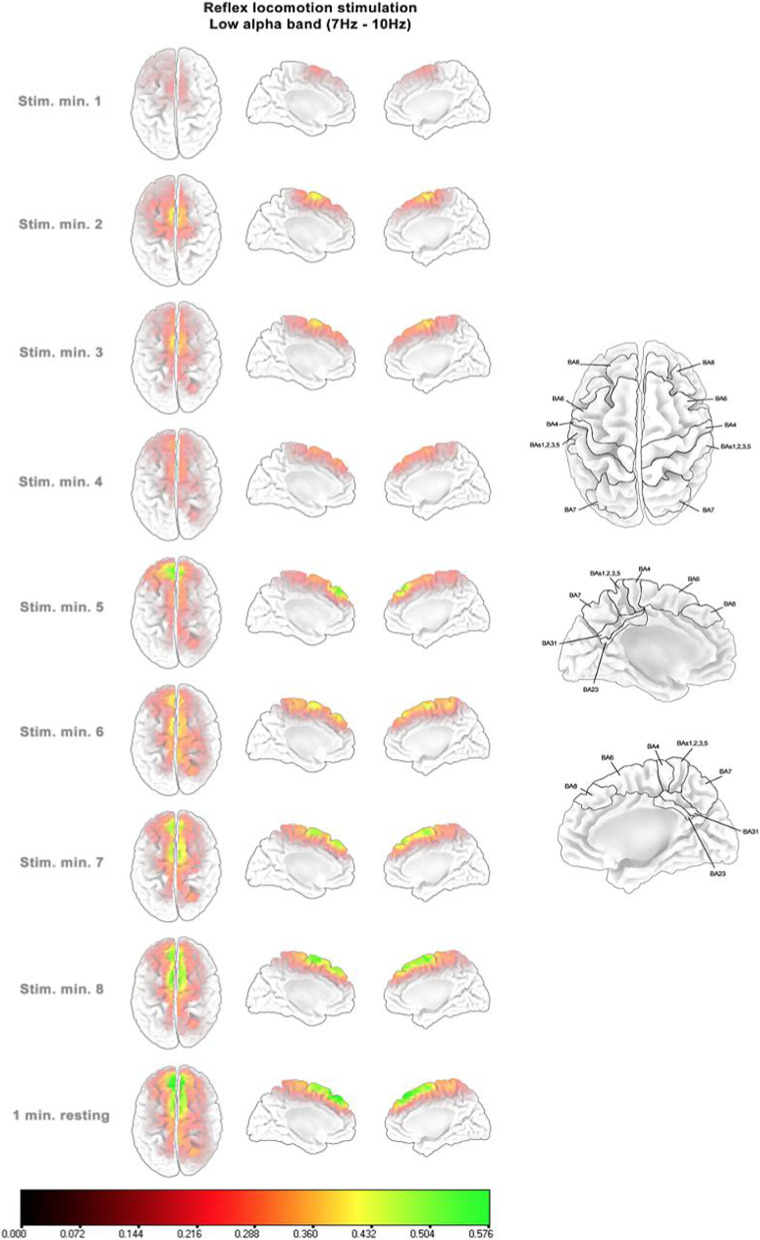
Standardized source power differences with respect to the first resting minute in consecutive minutes of reflex locomotion stimulation at low alpha band (7–10 Hz) for the V-STI-group. Implicated Brodmann areas are sketched over the cortex template on the right, according to the LORETA-KEY software

**Fig. 6:**
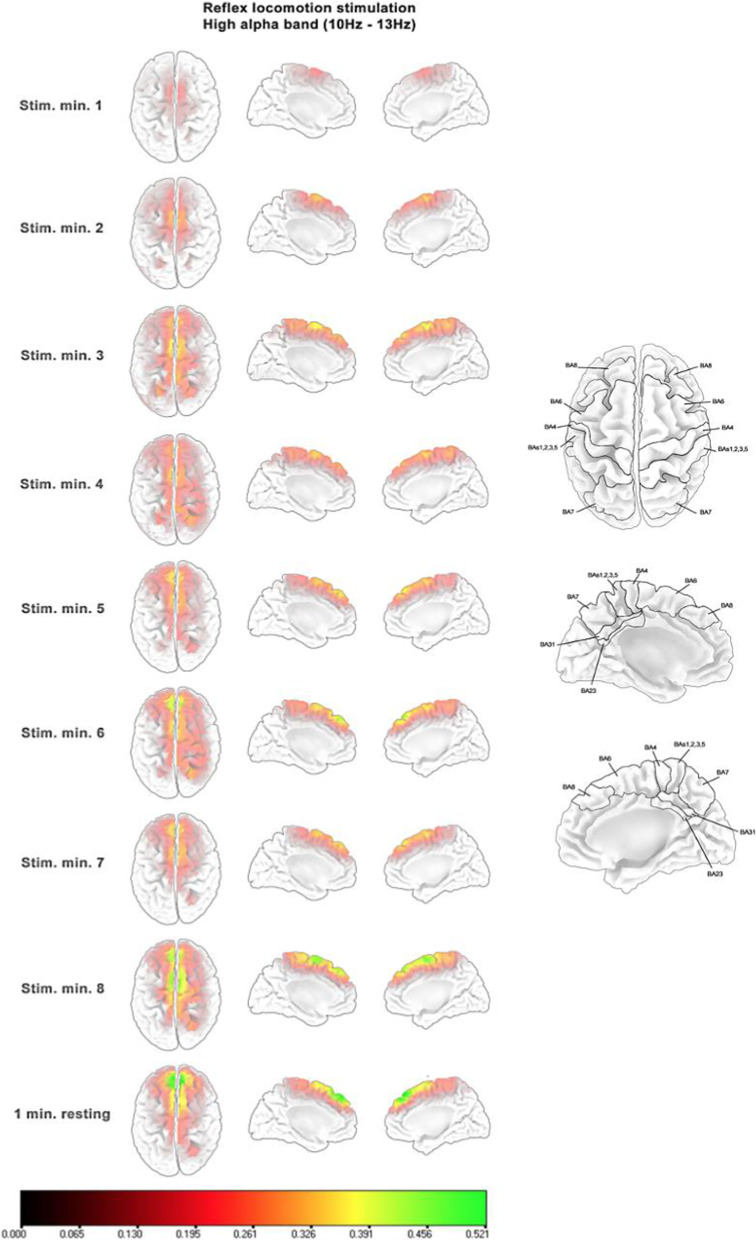
Standardized source power differences with respect to the first resting minute in consecutive minutes of reflex locomotion stimulation at high alpha band (10–13 Hz) for the V-STI-group. Implicated Brodmann areas are sketched over the cortex template on the right, according to the LORETA-KEY software

Figures 4, 5 and 6 show the standardized source power difference with respect to the first resting minute of each consecutive minute of reflex locomotion stimulation and the last resting minute for the frequency bands that presented statistically significant differences reported in the previous section, only for the V-STI group. All frequency bands presented an initial bilateral activation of the superior and medial SMA (BA6) during the first minute. This activation was maintained until the fourth minute. During the fourth minute, the activation decreased in the three frequency bands. From the fifth minute, the activation in the superior and medial SMA rose again in the three frequency bands. However, the theta band (Fig. 4) also presented an increasing bilateral activation of the superior and medial primary motor (M1) and somatosensory (S1) areas, as well as of the superior and medial parietal areas (BA5, BA7), whereas the low alpha and high alpha bands (Figs. 5 and 6, respectively) also presented an increasing bilateral activation of the more frontal pre-SMA (BA8). In the three frequency bands, the maximum level of activation was reached in the last minute of stimulation, keeping this level during the last resting minute under no stimulus.

## Discussion

Our findings highlight that the specific stimulation area at intercostal space, on the mammillary line between 7 and 8th ribs according to Vojta therapy differentially increased bilateral activation in SMA (BA6) and Pre-SMA (BA8), BA5, BA7, BA23 and BA31 in the theta, low and high alpha bands in healthy subjects. However, a cortical activation was registered in both stimulation groups. In our best knowledge, cortical activity assessed by EEG generated by specific tactile stimuli, under the locomotor stimulation paradigm had not been studied so far.

Irrespective of the cortical activation site, our results confirmed that a sustained pressure stimulation produced a cortical activation which agreed with previous fMRI studies [11, 12] that as well described a bilateral cortical activation at V-STI-group concordant with our results. However, EEG allows studies of much longer stimulation periods than fMRI due to it is less sensitive to movement artifacts when the stimulation triggers them and the time of the therapist applying the stimulation in the fMRI room is very limited, but unlimited with EEG.

In addition, the cortical areas activated by the reflex locomotor stimulation are also in agreement with previous studies [10–12], which suggest that sustained pressure stimulation applied on Vojta therapy [14] produces changes in fMRI in healthy subjects cortex compared to control group, concretely at upper parietal lobe and premotor cortex. Moreover, in our study, an increasing bilateral activation of the superior and medial primary motor (M1) and somatosensory (S1) areas is showed on V-STI-group after 5 min of sustained pressure. Similar activations were also previously described by Hok [12], where a statistically significant higher neuronal activity at M1 on stimulation V-STI-group was found.

Given the reflex nature of the movement evoked by the pressure stimulation used in our study, the activation of motor-related cortical areas, especially SMA, pre-SMA and PMA, might seem implausible. SMA is a cortical structure significantly related to planification and motor execution. However, it is also involved in the automatic movement’s initiation [14, 34, 35], evidenced the relationship between this area and the internal motor programs activation for the locomotion initiation. Pre-SMA rostral part seems to participate at planification process while its caudal portion at motor execution [36–38]. Besides, both areas, SMA and pre-SMA, are also linked to other structures as the superior frontal gyrus, thalamus, putamen and cerebellum [39–41]. Motor control is mostly related by all these structures [42] whose interaction during tactile and proprioceptive stimulation on pectoral site was already suggested by Sanz et al. [10], describing its high activation’s component of automatic locomotion paths on Vojta therapy. In the present study, a significant SMA (BA6) and pre-SMA (BA8) activation increasingly appeared during reflex locomotion stimulation, distinctly activated during minutes 4 to 8.

Previous research on proprioceptive stimulation over skin surface and tendon showed a motor network activation which involves M1 area, PMA and SMA, concurred with ours results [43]. Indeed, results are along the lines of previous research (Sanz el al.) at basal ganglia participation [44] and cerebellum together with SMA as structures participating in the intention of movement [45]. These findings highlight the potential of sensorial stimuli's importance for central nervous system activation for planification and movement feedforward. Moreover, Naito et al. [46] revealed the importance of M1 area and premotor area at the somatic perception of movement. According to these results, our results also showed a bilateral activation from minute 5 at primary cortex M1 and somatosensorial area S1. This activation is produced on a synchronic way related to association areas and SMA activation. In accordance with Naito et al. [43], our results propose a motor and sensorial network activation’s existence during sensorial, tactile, and proprioceptive stimuli.

Superior parietal areas also play a role in movement execution. In our work, as a result of the sensorial stimuli at pectoral site, a superior parietal cortex (BA5, BA7) activation is produced. Association’s neurons from BA5 area are capable of integrate the tactile and proprioceptive information for global posture pattern model [47]. At this association area, stimuli are registered and integrated expanding its information to other subcortical areas. [48]. This somatic information is essential to body schema awareness and spatial perception. Body schemata in the human brain revealed by kinesthetic illusions were evidenced to provide essential contributions to corporeal awareness and motor control [43]. In addition, the posterior parietal brain areas significantly activated in the V-STI-group covered the fronto-parietal action-observation network (AON), which relies on the body representation in the brain and is highly involved during automatic motor anticipation, for example in sport practice [49, 50].

Finally, the areas that presented statistically significant difference with respect to the first resting minute in the V-STI-group in our study are compatible with the findings from classical experiments for functional mapping by cortical electrical stimulation in the *Cercopithecus* monkey [51] and humans [52], and by postmortem injury analysis in humans [53]. The former studies converge in the decisive role of superior and medial Pre-SMA, SMA and, superior and medial parietal areas (BA5, BA7) in the execution of eyes, head and trunk movement to the contralateral direction and the synergic contraction of contralateral extremities. This motor behavior is a subset of the one elicited by reflex locomotion stimulation [14].

Regarding the cortical activity generated by tactile stimuli, no studies have been found that evaluate it in isolation using EEG, so no comparisons can be made regarding cortical activation in the different frequency bands. With respect to the theta band differences in the control group, they are compatible with the characteristic midline synchronization of theta waves during the transition from wakefulness to sleep [54–56], which was the observed behavior in most participants in this group.

Our research presents several limitations. First, the source power of the brain activity was calculated from 32 electrodes. This number makes the source localization of cortical activity less accurate than a higher number of electrodes. However, the localization error distance (LED) is comparable to more dense electrode configurations especially for upper brain areas (57), as the ones presenting significant differences in the present study. Next, the movement response intensity in the V-STI-group was heterogeneous, as expected in Vojta’s stimulation in adults [14], with some participants even showing no movement response at all. This heterogeneity was not taken into account in the analysis. In addition, we only recorded one minute after the stimulation intervention. During this minute, the cortical activation effects reached in the last minute of stimulation kept. This points to longer effects of the intervention, but we cannot determine or estimate how longer with our study design. Besides, this study was carried out with a relatively small sample of healthy subjects. This implies that, despite consistent changes observed, our results could not be extrapolated to patients with neurological disorders or other conditions. Future studies should be conducted with groups of patients and a sample-matched control group to avoid observer's bias. Moreover, the analysis of the proposed assessment would also be relevant linked to clinical and observational tests to understand functional changes due to tactile stimuli under the locomotor stimulation in patients with neurological disorders.

## Conclusion

A specific sensorial and proprioceptive stimulation at intercostal space, on the mammillary line between 7 and 8th ribs according to Vojta therapy evoke cortical great influence areas (SMA and Pre SMA) activation on planification and motor execution. The specific sustained pressure stimulation produced a sensorimotor system activation, bilaterally at premotor (BA6, 8) area and motor (M1) area, even one minute after the stimulation. These findings might denote an innate automatic motor component which is triggered after stimulation of the pectoral area, in accordance with that described in Vojta Therapy. Indeed, posterior cingulate cortex activation might confirm the importance on the stimuli at the motor answer execution. Further studies should corroborate cortical activation of innate motor paths and its implementation of automatic circuits in patients with neurological disorders, as well as comparing the results of longer and shorter duration stimulations to determine effective stimulation periods when applying the therapy.

## Data Availability

The data that support the findings of this study are available from the corresponding author upon reasonable request.

## Abbreviations

fMRI: Functional magnetic resonance imaging
EEG: Electroencephalographic
non-STI-group: Non-specific tactile input-group
V-STI-group: Vojta specific tactile input-group
CONSORT: CONsolidated Standards of Reporting Trials
ASR: Artifact subspace reconstruction
FIR: Finite impulse response
ICA: Independent component analysis
MARA: Multiple artifact rejection algorithm
SMA: Supplementary motor area
PMA: Premotor area
BA: Brodmann areas
AON: Action-observation network
LED: Localization error distance
